# Genetic editing of HLA expression in hematopoietic stem cells to broaden their human application

**DOI:** 10.1038/srep21757

**Published:** 2016-02-23

**Authors:** Hiroki Torikai, Tiejuan Mi, Loren Gragert, Martin Maiers, Amer Najjar, Sonny Ang, Sourindra Maiti, Jianliang Dai, Kirsten C. Switzer, Helen Huls, Gladys P. Dulay, Andreas Reik, Edward J. Rebar, Michael C. Holmes, Philip D. Gregory, Richard E. Champlin, Elizabeth J. Shpall, Laurence J. N. Cooper

**Affiliations:** 1 Division of Pediatrics, The University of Texas MD Anderson Cancer Center, Houston, TX, U.S.A; 2 Bioinformatics Research, National Marrow Donor Program, Minneapolis, MN, U.S.A; 3 Department of Biostatistics, The University of Texas MD Anderson Cancer Center, Houston, TX, U.S.A; 4 Sangamo BioSciences, Inc., Richmond, CA, U.S.A; 5 Department of Stem Cell Transplantation and Cellular Therapy, Division of Cancer Medicine, The University of Texas MD Anderson Cancer Center, Houston, TX, U.S.A; 6 Ziopharm Oncology, Inc., Boston, MA, U.S.A.

## Abstract

Mismatch of human leukocyte antigens (HLA) adversely impacts the outcome of patients after allogeneic hematopoietic stem-cell transplantation (alloHSCT). This translates into the clinical requirement to timely identify suitable HLA-matched donors which in turn curtails the chances of recipients, especially those from a racial minority, to successfully undergo alloHSCT. We thus sought to broaden the existing pool of registered unrelated donors based on analysis that eliminating the expression of the HLA-A increases the chance for finding a donor matched at HLA-B, -C, and -DRB1 regardless of a patient’s race. Elimination of HLA-A expression in HSC was achieved using artificial zinc finger nucleases designed to target HLA-A alleles. Significantly, these engineered HSCs maintain their ability to engraft and reconstitute hematopoiesis in immunocompromised mice. This introduced loss of HLA-A expression decreases the need to recruit large number of donors to match with potential recipients and has particular importance for patients whose HLA repertoire is under-represented in the current donor pool. Furthermore, the genetic engineering of stem cells provides a translational approach to HLA-match a limited number of third-party donors with a wide number of recipients.

Transplantation of allogeneic hematopoietic stem cells (HSC) into recipients with hematologic disorders reconstitutes normal hematopoiesis and gives rise to the graft-versus-tumor effect. The success of allogeneic hematopoietic stem-cell transplantation (alloHSCT) depends on the extent of matching of classical class I and II HLA alleles between a particular donor and their recipient, as disparate HLA molecules are targets for cellular- and antibody-mediated immune responses. This can compromise the therapeutic effect as manifested by graft-versus-host-disease (GVHD) and/or graft failure. Engrafted T cells mediating the GVL-effect recognize major and minor histocompatibility antigens which can be shared on recipients’ normal cells. Thus, GVHD that does not threaten survival, can favor the outcome of the patient with cancer. Graft failure is typically caused by patients’ resident humoral and cellular immune responses, including T-cells and NK cells, which recognize and eliminate infused HSCs^1^.

The HLA cluster on chromosome 6p21 is among the most polymorphic region in the human genome, yet haplotypes are conserved due to the relatively rare occurrence of linkage disequilibrium in this region^2^. Thus, the best-case scenario for a recipient requiring alloHSCT is about 30% based upon finding a first-degree relative that is at least matched at HLA-A/B/DRB1 taking into account that patients often have more than one sibling. When such donors are unavailable, recipients may benefit from over 10 million adult volunteers registered with the National Marrow Donor Program (NMDP) and for whom the repertoire at HLA/A/B/C/DRB1 are known. At least 7 of these 8 recorded HLA alleles need to be matched to safeguard the recipient^3^,^4^ resulting in insufficient numbers of donors to meet the current needs of potential recipients. As the number of recipients is expanding in excess of the number of suitable donors this asymmetry will further reduce the ability of future to undergo alloHSCT^5^.

Unrelated umbilical cord blood (UCB) is an alternative source of alloHSC with a less stringent need to match HLA types as compared with harvesting HSC from bone marrow or non-neonatal peripheral blood. However, failure to restore hematopoiesis after allogeneic UCB transplantation due to HLA-specific antibodies in the recipient^6^,^7^,^8^ and the small number of recoverable cells from UCB undermines the potential for therapeutic success. In addition, these complications can be exacerbated by the degree of HLA-mismatch between the UCB donor and recipient^9^. E*x vivo*-propagated UCB may increase the chance to undertake allogeneic UCB transplantation in adult patients, but differentiation of HSC may occur during expansion and lead to the engraftment of a subset of differentiated cells unable to completely restore hematopoiesis^10^,^11^,^12^,^13^,^14^.

Another alternative approach for allogeneic HSCT is to infuse haploidentical HSC. This strategy requires the identification of a 3 or 4 HLA-matched related donor to the benefit of nearly all potential recipients who can find such donors such as from parents. Indeed, selection of maternal over paternal donor results in the better survival^15^. Recent improvement in therapy-related mortality have raised the enthusiasm that haploidentical HSCT may be the preferred approach for those who cannot find an HLA matched donors. However, the incidence of graft failure and GVHD remain barriers to therapeutic success^16^.

We therefore sought to develop an alternative approach to increase the chance for finding HLA-matched donor without the need and expense to recruit additional potential donors based upon the application of an artificial nuclease to introduce double strand breaks (DSB) within the HLA complex. These DSB will be preferentially healed by error-prone non-homologous end joining repair pathway which leads modified cells to lose expression of the targeted gene. We chose to employ a zinc finger nuclease (ZFN)^17^ for our studies since these proteins have been validated as safe and efficacious in an early-phase clinical trial infusing T cells genetically edited to prevent CCR5 expression as investigational treatment of patients with HIV^18^. Furthermore, we had undertaken a preliminary study demonstrating that embryonic stem cells can be genetically edited with ZFN to eliminate HLA-A expression^19^.

Our analysis of NMDP data revealed that just the elimination of HLA-A expression increases the probability for finding a suitable donor matched at HLA-B/C/DRB1. Indeed, the disparity at HLA-A particularly decreases the chance for a potential recipient from racial minority finding a donor as their HLA repertoire is not well-represented in the current donor pool. We demonstrated that HLA-A expression can be eliminated from HSC using ZFN dimers designed to target DNA encoding HLA-A alleles. We further showed that HLA-A^neg^ HSCs maintain their ability to engraft in immunocompromised mice and reconstitute human hematopoietic cells. This strategy will increase the probability for finding suitable HLA-matched HSCs for patients who cannot find suitable biological product in the current unrelated donor registry. This approach also has implications for sourcing HLA-matched cells from third-party “universal” donors as a limited pool of allogeneic HSC can be bio-engineered to service multiple recipients.

## Materials and Methods

### Study approval

UCB were obtained from the bank at MD Anderson Cancer Center (MDACC) prior to cryopreservation in accordance with the Declaration of Helsinki and consents were approved by the Institutional Review Board of MDACC.

### Analysis of NMDP registry data

8,994,658 adult donors with HLA data were registered with NMDP as of December 2010. The race categories of donors were self-reported and collected on standardized forms. For alloHSCT engrafting HSC from non-neonatal donors, an HLA-match between donor and recipient was based on allele-level matching at HLA-A/B/C/DRB1 loci, designated as 8/8 HLA-matching. A single allele mismatch at any of these loci is designated as 7/8 HLA-matching. When considering HLA disruption, one locus at a time was removed from mathematical consideration, so comparable definitions were identified as 6/6 and 5/6 HLA-matching. HLA haplotype frequencies for the 21 racial population groups were calculated from DNA-typed registry donors using the expectation-maximization algorithm^20^. Four-locus allele-level haplotype frequencies (HLA-A/B/C/DRB1) were used as the baseline for non-neonatal donors. Three-locus HLA-B/C/DRB1 haplotype frequencies were generated by summing over the HLA-A locus^21^. The effective number of adult donors was calculated by multiplying the number of donors in each populations by the race-specific donor availability rates. The haplotype frequencies and number of effective adult donors for each population were put into a population genetic matching model that assumes Hardy-Weinberg equilibrium among haplotypes and genotypes^22^. The model calculated the population-specific HLA-match likelihoods at varying match stringencies for the given registry size^21^. The accepted clinical standards for unrelated donor transplantation in the USA are based on identifying 7/8 HLA-matched and 8/8 HLA-matched adult donors^23^. For the models considering HLA disruption, comparable 5/6 and 6/6 matching were computed.

### Design of ZFNs targeting HLA-A and template plasmid and *in vitro*-transcription of messenger RNA

The target sites and design of HLA-A targeting ZFNs have been described^19^. We cloned the ZFN construct from pVAX plasmid and expressed it in pGEM4z/A64 plasmid (kindly provided by Dr. E. Gilboa, University of Miami)^24^ as shown in Supplementary Fig. 1. The DNA template plasmids were linearized with *SpeI* and *in vitro*-transcription was performed (T7 MEGA Script: Ambion) according to manufacturer’s instructions.

### Isolation, electroporation, and culture of HSC

Mononucleocytes (MNCs) were isolated by Ficoll gradient separation from UCB within 24 hours of collection at time of delivery. Lineage (Lin)^neg^CD34^+^ cells were isolated by using CD34 diamond isolation kit (Miltenyi Biotec) according to the manufacturer’s instruction. Lin^neg^ CD34^+^ cells were cultured in StemSpanH3000 (Stemcell Technologies) containing FLT-3L, TPO, and SCF overnight. Cells were then collected and spun at 300 g for 5 minutes. After aspirating supernatant, cells were resuspended with 100 μL of Nucleofector CD34 kit (Lonza). Just before electroporation, cells were mixed with the two *in vitro*-transcribed mRNA and transfered to a cuvette. Nucleofection was performed using the Nucleofector II device with program U-008. Immediately after electropotation, cells were recovered in StemSpanH3000 media. Two hours after incubating cells in 37 °C incubator, cytokines (FLT-3 L, TPO, SCF [each 100 ng/mL], and IL-6 [50 ng/mL] with or without 1 μM SR-1) were added to the culture and maintained in 37 °C and 5% CO_2_.

### Flow cytometry

The antibodies used in this study can be found in Supplementary Table 1. Data was acquired on a FACS Calibur using CellQuest version 3.3 (BD Biosciences) and analyzed by FlowJo version 10.1 (Tree Star, Inc).

### Mutation detection assay

Nuclease activity was determined by the Surveyor nuclease assay (Transgenomic) according to the manufacture’s instructions^25^. The PCR primers used for the amplification of target were; Forward; 5′-GGGTCCGGAGTATTGGGACGG-3′ Reverse; 5′-TTGCCGTCGTAGGCGTACTGGTG-3′

For the next generation sequencing, PCR reactions were performed with a locus-specific primer pair containing adaptor sequences. PCR products were diluted (1:200 in H_2_O) and used in a 10 μL PCR reaction to add the Illumina library sequences and sample-specific barcode sequences. PCR products are then pooled and sequenced on an Illumina MiSeq Instrument (Illumina) with a v2 300 cycle sequencing kit using paired-end 150 bp reads. Sequences in FASTQ format from the MiSeq were demultiplexed based on the barcode reads. Paired-end reads were then combined and adapter trimmed using SeqPrep (J. St. John, unpublished, https://github.com/jstjohn/SeqPrep). Reads that do not pair were discarded. Paired reads were filtered for those that contain perfect matches to the 5′ and 3′ terminal 23 bp of the expected amplicon to eliminate low levels of off-target amplification, primer-dimers, and primer synthesis errors. Paired reads were then aligned to the expected wild-type amplicon and scored as an indel if gaps are present in the alignment. ZFN-derived activity was assessed by determining the fraction of total paired sequence reads per sample that contain indels in the nuclease-treated cells. This value was then compared to the same value obtained from the non-ZFN treated control sample amplified with the same locus-specific primers.

### Gene expression analysis

Total RNA from fresh HSCs or cultured HSCs was isolated by Allprep DNA/RNA Micro Kit (Qiagen). Integrity of RNA was validated by Bioanalyzer (2100 Expert, Agilent). Gene expression analysis was undertaken using the HumanHT-12 v4 Expression BeadChip (illumina). The data were 2-based logarithm-transformed and the linear mixed model was used to reveal the overall association between the abundance of mRNA and conditions used to culture HSC. If the overall association was significant (p-value < 0.01), then pairwise comparisons using Tukey’s post hoc tests were performed to detect the difference in expression between treatments. An adjusted p-value < 0.01 and fold change > = 2 or < = −2 were considered statistically significant. PCA was used to visualize the similarities and variations among samples. Sample clustering and heat map analyses were performed using R (version 3.0.2, R Foundation) and the program dChip^26^. We deposited the data at the Gene Expression Omnibus (GEO) public repository under the accession number GSE67093.

### *In vitro* colony forming assay

HSCs that were electro-transferred with mRNA coding for ZFN were cultured overnight and 1,000 cells were diluted in 3 mL of semi-colloid culture medium (Methocult H4435, Stemcell Technologies) and distributed within a 6-well plate. Twelve days later, colonies were counted and plucked for analysis under inverted microscope.

### *In vivo* experiment

Experiments were approved by the Institutional Animal Care and Use Committee at MDACC and performed in accordance with the guidelines and requirements set forth by the Public Health Service (PHS) Policy on Humane Care and Use of Laboratory Animals, the U.S. Department of Health and Human Services Guide for the Care and Use of Laboratory Animals, and the USDA Animal Welfare Act. Five to six week old female NSG mice (Jackson laboratory) were irradiated to 175 cGy. The day after, 10^6^/mouse *ex vivo*-propagated HSC in PBS were injected via tail vein. Mice were monitored human cell engraftment by serial sampling of PB every 4 weeks. At 16 weeks, mice were sacrificed and cells were obtained from PB, spleen, and BM for flow cytometry. Statistical significance were analyzed by multiple t-test with Holme-Sidak method using Graph-Pad Prism 6 (GraphPad Software, Inc.).

## Results

### Impact of disrupting one HLA locus on the chance for finding an HLA-matched unrelated HSC donor

**Figure 1:**
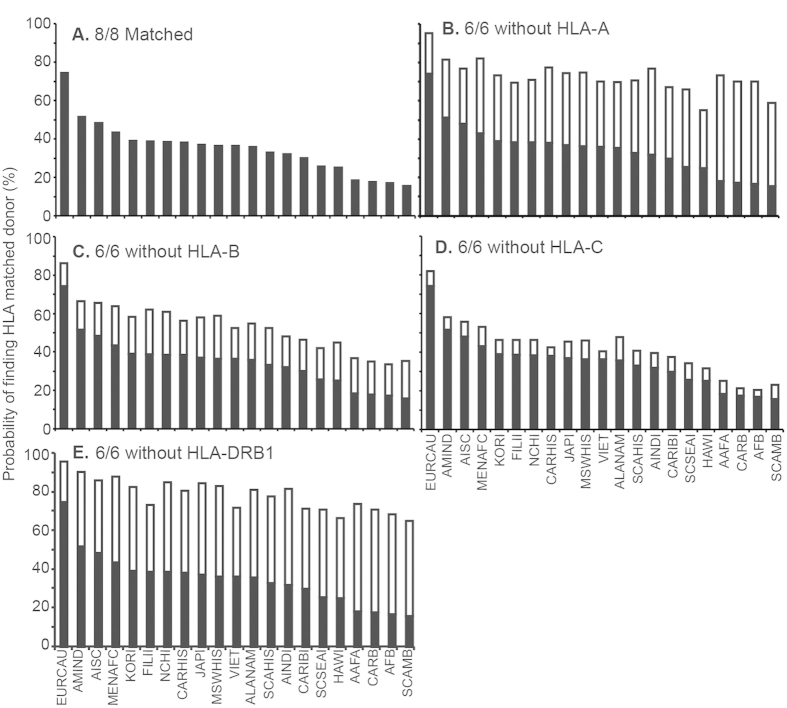
Calculated impact of eliminating an HLA allele from consideration on increasing the chance for finding a suitable HLA-matched unrelated allogeneic donor registered with NMDP (**A**) Probability of finding a donor matched at 8/8 HLA alleles (HLA-A/B/C/DRB1) in each race group (Supplementary Table 1). Probability of finding a donor matched at 6/6 HLA alleles in each ethnic race group. considering: (**B**) HLA-B/C/DRB1, (**C**) HLA-A/C/DRB1, (**D**) HLA-A/B/DRB1, and (**E**) HLA-A/B/C. Open bar shows the increase in the probability of finding HLA matched donors.

We evaluated the influence of finding an HLA allele-matched unrelated donor if one HLA locus was eliminated from consideration in the NMDP registry based upon typing for HLA/A/B/C/DRB1. In particular, we were interested in whether this would improve the probability for finding HLA-matched unrelated allogeneic donor for recipients of racial minorities as previous reports have implicated that successful matching was dependent on racial background of the recipients^21^,^27^,^28^. Twenty one races (Supplementary Table 2) and associated HLA data were retrieved for 8,994,658 adult donors registered with NMDP. European Caucasians have the highest chance for finding 8/8 HLA-matched donors while potential recipients with Black South/Central American backgrounds have the lowest probability of finding an 8/8 HLA-matched donor (Fig. 1A). We analyzed the effect of eliminating one HLA locus for the increase of finding HLA-matched donors in each race group. Eliminating the need for matching at HLA-A or HLA-DRB1 significantly increases the chance for finding HLA-matched donor from the registry compared to elimination of HLA-B or HLA-C alleles (Fig. 1B–E). The improved chance of identifying a potential unrelated allogeneic donor was especially significant in racial minorities which currently have a lower chance for locating an 8/8 HLA allele-matched unrelated allogeneic donor. Based on similar probability calculations, we also demonstrated that allowing a one allele mismatch between donor and recipient after elimination of one HLA allele at A, B or DRB1 loci from consideration increased the probability of finding an HLA-matched donor to over 90% in all races (Supplementary Fig. 2). In summary, we have revealed that among potential donors registered with NMDP, heterogeneity at HLA-A and HLA-DRB1 loci disproportionately undermines the chances of a given recipient to identify an unrelated 8/8 HLA allele-matched donor for allogeneic HSCT. Thus, we found that eliminating the need to harmonize at these two loci significantly increases the probability of finding a suitable donor, especially in minority racial groups.

### HLA-A in HSC as a target for designer ZFN

Although the elimination of HLA-DR has a similar calculated benefit as elimination of HLA-A to improve the chance of a recipient finding an HLA-matched allogeneic donor, previous reports have shown that HLA-DR is not well expressed on primitive multi-potent HSCs^29^,^30^. We confirmed that HLA-DR expression on CD34^pos^ cells was heterogeneous, especially in CD34^+^CD38^neg^ sub-population, where primitive HSC were enriched (Supplementary Fig. 3). This led us to focus on eliminating the expression of HLA-A alleles. We previously designed a pair of ZFN targeting exon 3 of the HLA-A gene^19^ (Supplementary Fig. 1). We reported that elimination of HLA-A2 expression in T-cells was complete after sorting, but only tested the impact of the ZFN species on a subset of HLA-A alleles. We therefore undertook ELISA-based screening to evaluate the binding specificity of the ZFN pair to target other HLA-A alleles. We demonstrated that the ZFN can efficiently bind to DNA coding for all common HLA-A alleles, except for HLA-A26 (Supplementary Table 3). These data demonstrate that targeting multiple HLA-A alleles using ZFN is feasible and a step towards the generation of allogeneic edited HSC for broad application.

### ZFN can eliminate expression of HLA-A in HSC

**Figure 2:**
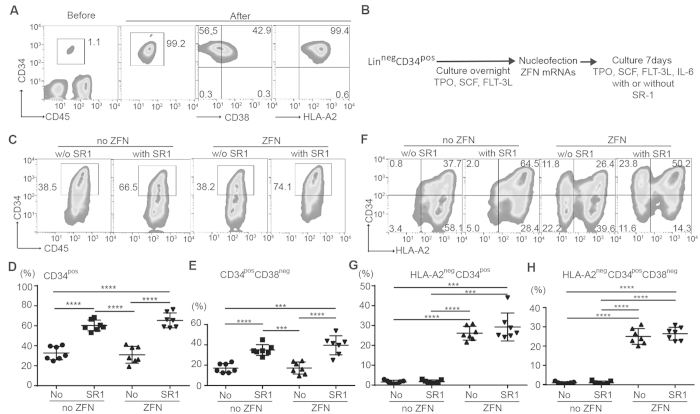
*Ex vivo* numeric expansion and genetic editing of HSC (**A**) Isolation of Lin^neg^CD34^+^ cells from UCB. Representative flow-cytometry data (n = 7) before and after selection with magnetic beads are shown. The number in the figure represents the percentage of each population. (**B**). Protocol to introduce ZFN targeting HLA-A as expressed from *in vitro*-transcribed mRNA. (**C**) Representative data (n = 7) for CD34 expression after seven days of *ex vivo* culture. (**D**) Distribution of percentage of CD34^+^ cells after the *ex vivo* culture. (**E**) Distribution of percentage of CD34^+^ CD38^neg^ cell after the *ex vivo* culture. ***p value < 0.001, ****p value < 0.0001. (**F**) Representative data (n = 7) for disruption of HLA-A expression on HSC.(**G**) Distribution of percentage of HLA-A2^neg^ cells within CD34^+^ cells after seven days of *ex vivo* culture. (**H**) Distribution of percentage of HLA-A2^neg^ cells within CD34^+^ CD38^neg^ cells expression after the *ex vivo* culture. Statistical significance were analyzed by multiple t-test with Holme-Sidak method using Graph-Pad Prism 6 (GraphPad Software, Inc.).

**Figure 3:**
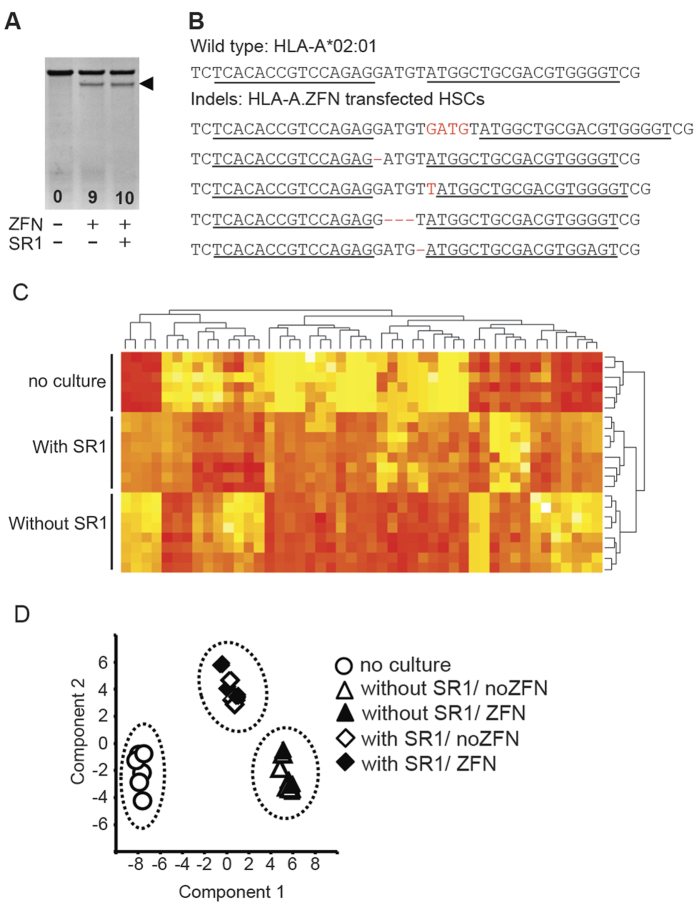
ZFN mediated HLA-A disruption (**A**) Representative (n = 7) mutation detection assay. Arrow shows the *Cel1*-digested band. Number indicates percentage disruption calculated by densitometry. (**B**) Top 5 indels induced by ZFN targeting HLA-A. Underlining reveales sequences targeted by the zinc finger binding domain. Red letters reveal indels. (**C**) Cluster analysis of gene expression based on R script and (**D**). PCA of gene expression using 47 differentially expressed genes in cells cultured with SR1 and cells cultured without SR1. The PCA revealed that the 1^st^ and 2^nd^ principal components accounted for 83% of the variance.

To evaluate HLA-A expression in HSC, we isolated a Lin^neg^ CD34^+^ sub-population from fresh UCB. The purity of CD34^+^ cells was over 99% and contained an average of 52% (standard deviation (SD) = 18%; n = 7) of CD38^neg^ cells. The enriched HSC expressed HLA-A on the cell surface which enabled us to detect disruption of expression by flow cytometry (Fig. 2A). After overnight stimulation of HSC, *in vitro*-transcribed mRNA encoding a ZFN pair were introduced by electroporation. These cells were then cultured with or without the aryl-hydrocarbon receptor antagonist, StemRegenin 1 (SR1), which has been reported to maintain HSC functionality^31^ (Fig. 2B). Seven days after *in vitro* culture, both with or without SR1, there was no difference in increase of total cells numbers, but the percentage of cells maintaining CD34 expression (and CD34^pos^CD38^neg^ phenotype) was significantly higher in those cells cultured in the presence of SR1 (Fig. 2C–E). As a result, the number of CD34^+^ cells (and CD34^+^CD38^neg^ cells) was significantly higher in the presence of SR1(11.86 ± 2.549 -fold [average ± SD, n = 7] in cells cultured with SR1 and 5.617 ± 0.7959 -fold [average ± SD, n = 7] in cells cultured without SR-1). The efficiency of disrupting HLA-A expression by ZFN was not different in cells cultured with SR1 and in cells cultured without SR1 (Fig. 2F–H). Desired changes following ZFN treatment occuring within the target sites were confirmed by a mutation detection assay and deep sequencing (Fig. 3A,B). We then undertook a gene expression microarray analysis comparing the HLA-A^neg^ versus HLA-A^+^ cultured HSC to evaluate a posible impact of genetic editing with ZFN. 9,224 genes were found to be significantly associated with the five experimental conditions (no propagation, culture with or without SR1 [ZFN transfected or Mock transfected HSC]) using a linear mixed model. Among these genes, 41 of 47 were found to be differentially expressed in cells cultured with SR1 versus those without regardless of whether there was exposure to editing by ZFN (Supplementary Table 4). These 47 differentially-expressed genes in HSC organized into three clusters (controls, HSCs with SR1 and HSC without SR1) (Fig. 3D) which is shown in the plot of principal component analysis (Fig. 3E). Six genes were found to be differentially expressed in HSC edited with ZFN compared with unedited cells (Supplementary Table 5). However, analysis of samples did not reveal clustering among 6 genes (not shown). In summary, expression of HLA-A can be efficiently disrupted in HSC by ZFN without apparent deleterious impact to overall gene expression.

### ZFN-edited HSC form lineage-specific colonies *in vitro*

**Figure 4:**
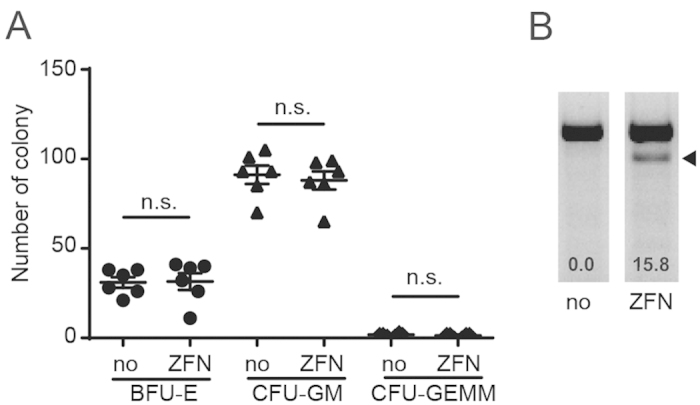
*In vitro* colony formation assay (**A**) Numbers of colonies formed after 12 days of culture. BFU-E: Burst forming unit erythroid, CFU-GM: Colony-forming unit granulocyte macrophage, CFU-GEMM: Colony-forming unit granulocyte erythrocyte monocyte macrophage, n.s.: not statistically significant. (**B**) Mutation detection assay of cells recovered from colonies. Number indicates percentage disruption calculated by densitometry.

We next evaluated the functional ability of HLA-A^neg^ cultured HSC to differentiate *in vitro* into multiple lineages of hematopoietic cells by colony-forming assay in semi-colloid culture medium. Twelve days after initiation of cultures, we obtained BFU-E, CFU-GM, and CFU-GEMM colonies from two sets of HSCs that were or were not edited with ZFN. Furthermore, we observed no apparent differences in the number of lineage-specific colonies between ZFN edited HSC and non-edited HSC (Fig. 4A). The mutation detection assay from differentiated colonies revealed genetic changes at the ZFN-targeting site only in ZFN edited HSC (Fig. 4B). These data confirm that the introduction of ZFN into cultured HSC does not inhibit their ability to differentiate into multi-lineage hematopoietic cells.

### ZFN-edited HSC can engraft and differentiate *in vivo*

**Figure 5:**
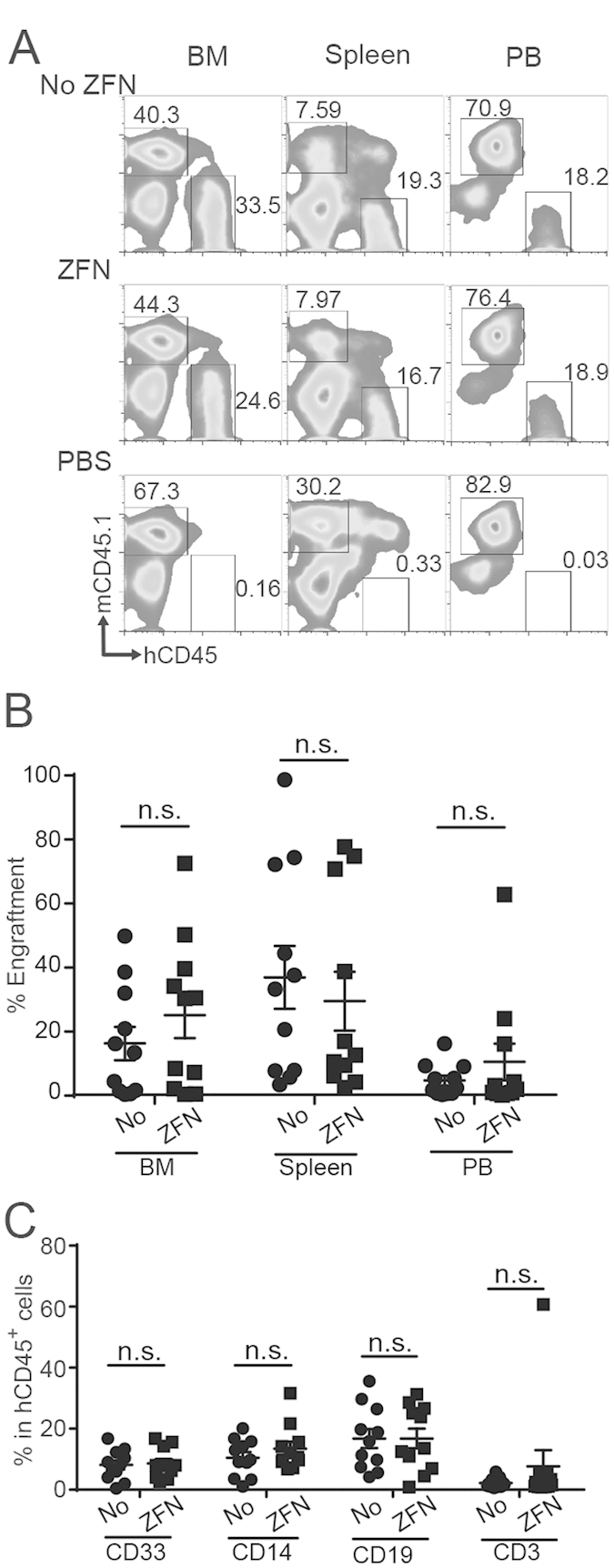
*In vivo* engraftment and differentiation of genetically edited HSC (**A)**. Representative (n = 11 in each group) flow cytometry data of HSC engraftment in NSG mice. BM: bone marrow, PB: peripheral blood. hCD45: human CD45, mCD45.1; mouse CD45.1. Each number represents percentage of corresponding rectangle area in total cells. (**B**) Percent engraftment of human cells in NSG mice. Percent engraftment was calculated by: 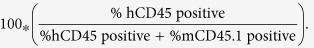
n.s.: not statistically significant. (**C**) Percentage of each lineage cells in total hCD45^+^ cells. CD33: CD33^+^CD14^neg^ myeloid cells, CD14: CD33^dull^CD14^pos^ monocytes, CD19: CD19^+^CD3^neg^ B-cells, CD3: CD19^neg^CD3^+^ T-cells.

**Figure 6:**
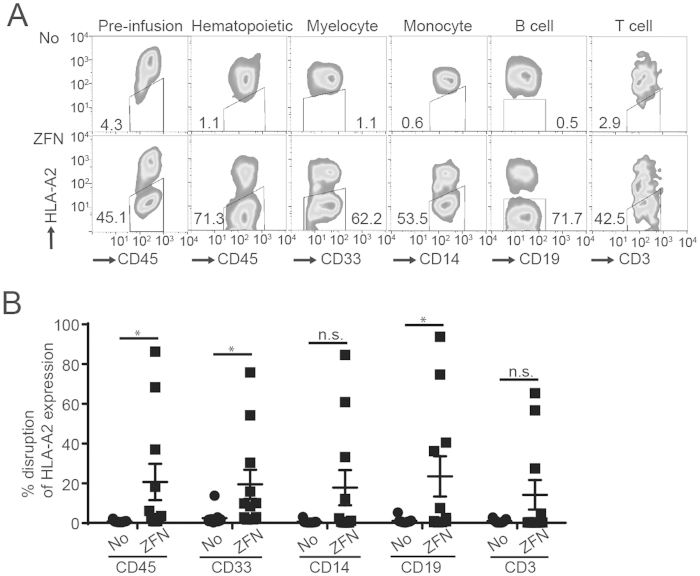
*In vivo* engraftment and differentiation of HLA-A^neg^ HSC (**A**) Representative (n = 11 in each group) flow cytometry data for HLA-A^neg^ population in each hematopoietic cell lineage. Numbers in figure represents percentage of HLA-A negative population. (**B**) Percent HLA-A^neg^ cells engrafted (n = 11 in each group). Error bar represents standard error. *p value < 0.05.

Next, we evaluated the ability of HLA-A^neg^ HSC to differentiate *in vivo* by injecting these edited and cultured cells into γ-irradiated female NSG mice. Sixteen weeks after injection, we demonstrated the engraftment of human cells in PB, BM, and spleen (Fig. 5A). The efficiency of engraftment within these tissues was not different between mice that received edited HSC versus the non-edited controls (Fig. 5B). The human cells present in BM were analyzed for expression of lineage-specific markers which revealed the desired differentiation of administed HSC into human myelocytes (CD33^+^CD14^neg^), monocytes (CD33^+^CD14^pos^), B-cells (CD19^+^), and T-cells (CD3^+^). Additionally, there were no apparent differences in the differentiation of sub-populations of cells from edited HSC versus the non-edited controls (Fig. 5C). The engrafted cells were analyzed for expression of HLA-A (Fig. 6A). This revealed the presence of HLA-A2^neg^ cells in human hematopoietic cells (20.46% HLA-A^neg^, n = 11 mice) as well as differentiated lineage-specific sub-populations (CD33^+^, CD14^+^, CD19^+^, and CD3^+^) (Fig. 6A,B). These data confirmed that ZFN can eliminate HLA-A expression from HSC which can engraft and differentiate in NSG mice thus revealing their multipotent potential.

## Discussion

We demonstrate that ZFN can be used to eliminate the expression of HLA-A alleles on HSC without adversely impacting their function. This has implications for broadening the application of allografts particularly for racial minorities who are candidates to undergo allogeneic HSCT from an unrelated donor. These data also begin to address the bio-engineeering of stem cells that can serve as a source of universal cells to achieve HLA-matched off-the-shelf therapies.

An analysis of the current NMDP donor registry revealed that the probability of finding an 8/8 HLA allele-matched unrelated donor was indeed associated with the race of patient and this is in agreement with prior reports^27^,^28^. Our modeling revealed that eliminating the need to match at HLA-A or HLA-DRB1 alleles disproportionately benefited the chance for finding an otherwise HLA-matched donor. This may reflect the genomic positioning of HLA-B and HLA-C alleles on 6p21 as they are relatively closely to each other (within 100 kb) reducing the recombination rate between these loci^2^. HLA-A and HLA-DRB1 alleles, however, are farther away (over 1 Mb) from the HLA-B/-C loci increasing the potential for recombination. We chose to eliminate HLA-A over HLA-DRB1 due to the expression level in primitive HSC(CD34^+^CD38^−^). An additional justification for targeting this allele is that as matching at HLA-DQB1 becomes more common, the high linkage disequilibrium between HLA-DRB1 and HLA-DQB1 would negate some of the advantage of removing HLA-DRB1.

The number of recipients undergoing unrelated alloHSCT using donor-derived products registered with the NMDP was 6,283 in 2013. This number has increased three-fold compared with 2003 and is expected to continue to increase (National Marrow Donor Program, a contractor for the C.W. Bill Young Cell Transplantation Program operated through the U. S. Department of Health and Human Services, Health Resources and Services Administration, Healthcare Systems Bureau. Donor Registry Transplant Data). However, not all potential recipients can currently benefit from alloHSCT due to a lack of finding a suitable donor, defined as matching at least 7 of 8 HLA alleles (HLA-A/B/C/DRB1). This is especially apparent for patients heralding from racial minorities as they face a disproportionate difficulty identifying an HLA-matched unrelated donor in the NMDP registry^32^. Indeed, of the 6,283 unrelated alloHSCT performed in the USA in 2013, only 1,003 were administered for recipients characterized as belonging to racial minorities. Within these 1,003 recipients, 34% of patients received cord blood and 66% received cell from adult donors. We estimate 12,000 patients in the US per annum would benefit from an unrelated alloHSCT and as 33% of the US population is considered to belong to a racial minority, it is calculated that 4,000 patients might benefit from allo-HSCT. This leaves approximately 3,000 recipients from racial minoriotes per year without a sutiable donor.

A widening of the unrelated and related donor pool has the greatest impact for minority patient populations and thus diseases which accumulate within a specific race. For example, sickle cell disease (SCD) with its autosomal recessive inheritance pattern is mostly seen in patients of African descent^33^. A problem for patients with clinically-severe SCD requiring alloHSCT is that only 14 to 18% of potential recipients are calculated to have an HLA-matched first-degree relative unaffected by SCD and thus be able to undergo the standard-of-care sibling alloHSCT^34^,^35^. For African Americans with any disease treatable by alloHSCT, but who lack an HLA-matched first-degree relative, the NMDP data reveal that only 17% of potential recipients can find an unrelated donor matched at HLA-A/B/C/DRB1. The chance for finding a suitable HLA-matched (at HLA-B/C/DRB1) donor for African Americans will substantially increase to 73% with our strategy to eliminate expression of HLA-A from HSC currently banked with NMDP.

Anti-viral immunity largely relies on the viral epitope presentation through diverse HLA allele on antigen presenting cells to T-cells. Thus, the engineered loss of HLA diversity may adversely affect T-cell immunity after HSCT as an individual heterozyogous for HLA has an apparent advantage over an individual homozygous for a given HLA in clearance of pathogen^36^. However, recent reports showed that epitopes presented on HLA class I can ehxibit promiscuous binding to other HLA class I^37^,^38^. This phenomenon is not limited to the so called super group of HLA, which shares similar peptide binding groove, but is also seen between different HLA loci. Therefore, these promiscuity of viral epitope presentation may maintain anti-viral immunity in patients receiving HLA-A disrupted HSCs.

We consider the genetic editing of HLA expression as step towards generating universal biological products defined as one donor’s cells suitable for sustained engraftment in multiple unrelated recipients. Complete elimination of HLA will likely sensitize the edited cells to elimination by resident NK cells. Enforced expression of non-classical HLA class I (*e.g*. HLA-E or HLA-G) may suppress such NK cell activation, but this will likely not completely avoid NK cell mediated killing of the infused cells^19^. The threshold for NK cell activation is maintained in part by inhibitory killer-immunoglobulin like receptors that bind to specific HLA-B or HLA-C alleles^39^. Thus, retaining these HLA loci on engrafted allogeneic cells bio-engieered to eliminate expression of HLA-A appears to be preferable, rather than complete elimination of HLA class I expression, in order to avoid NK cell mediated rejection. Another application of editing HLA-A-edited outside of the context of alloHSCT would be generate “off-the-shelf” CAR^+^ T cells or NK cells from allogeneic HSC^40^. Elimination of HLA-A expression from third-party T cells and NK cells will also increase the probability of matching remaining HLA alleles between a universal donor and recipients which will decrease the chance of immune-mediated rejection after administration and thus increase the chance of a therapeutic response.

In summary, elimination of HLA-A expression with an artificial nuclease increases the chance of finding an unrelated donor already registered with NMDP and matched at HLA-B/C/DRB1. The increased probability of a successful match after this genetic editing is especially pronounced for potential racial minority. We find that complete and permanent disruption of HLA-A expression in HSC can be achieved by ZFN without apparently adversely affecting the ability of these genetically edited and cultured progenitor cells to reconstitute hematopoiesis. This bio-engineering strategy is anticipated to increase the opportunity for recipients to undergo successful and sustained engraftment of stem cells and their differentiated progeny when the HLA diversity of potential donor cells is constrained.

## Additional Information

**How to cite this article**: Torikai, H. *et al*. Genetic editing of HLA expression in hematopoietic stem cells to broaden their human application. *Sci. Rep*. **6**, 21757; doi: 10.1038/srep21757 (2016).

## Supplementary Material

Supplementary Information
