# Supplementary material for: Genetic editing of HLA expression in hematopoietic stem cells to broaden their human application
Source: Sci Rep. 2016 Feb 23;6:21757. doi: 10.1038/srep21757 (PMC4763194; doi:10.1038/srep21757)
Supplement: Supplementary Information [file srep21757-s1.pdf]

SUPPLEMENTAL INFORMATION

Genetic editing of HLA expression in hematopoietic stem cells to broaden their human application

AUTHORS

Hiroki Torikai[1 *], Tiejuan Mi[1], Loren Gragert[2], Martin Maiers[2], Amer Najjar[1], Sonny Ang[1], Sourindra Maiti[1], Jianliang Dai[3], Kirsten C. Switzer[1], Helen Huls[1], Gladys P. Dulay[4] Andreas Reik[4], Edward J. Rebar[4], Michael C. Holmes[4], Philip D. Gregory[4], Richard E. Champlin[5], Elizabeth J. Shpall[5], and Laurence J.N. Cooper[1, 6 *]

AFFILIATIONS

[1] Division of Pediatrics, The University of Texas MD Anderson Cancer Center, Houston, TX

[2] Bioinformatics Research, National Marrow Donor Program, Minneapolis, MN

[3] Department of Biostatistics, The University of Texas MD Anderson Cancer Center, Houston, TX

[4] Sangamo BioSciences, Inc., Richmond, CA

[5] Department of Stem Cell Transplantation and Cellular Therapy, Division of Cancer Medicine, The University of Texas MD Anderson Cancer Center, Houston, TX

[6] Ziopharm Oncology, Inc., Boston, MA,

***CORRESPONDING AUTHORS (Co-Corresponding Authors)**

Laurence J.N. Cooper, M.D., Ph.D.

Division of Pediatrics, The University of Texas MD Anderson Cancer Center

Unit 907, 1515 Holcombe Blvd., Houston, TX 77030

Phone: (713) 563-3208; Fax: (713) 792-9832;

E-mail: ljncooper@mdanderson.org

Hiroki Torikai, M.D.

Division of Pediatrics, The University of Texas MD Anderson Cancer Center

Unit 907, 1515 Holcombe Blvd., Houston, TX 77030

Phone: (713) 792-8195; Fax: (713) 792-9832;

E-mail: htorikai@mdanderson.org

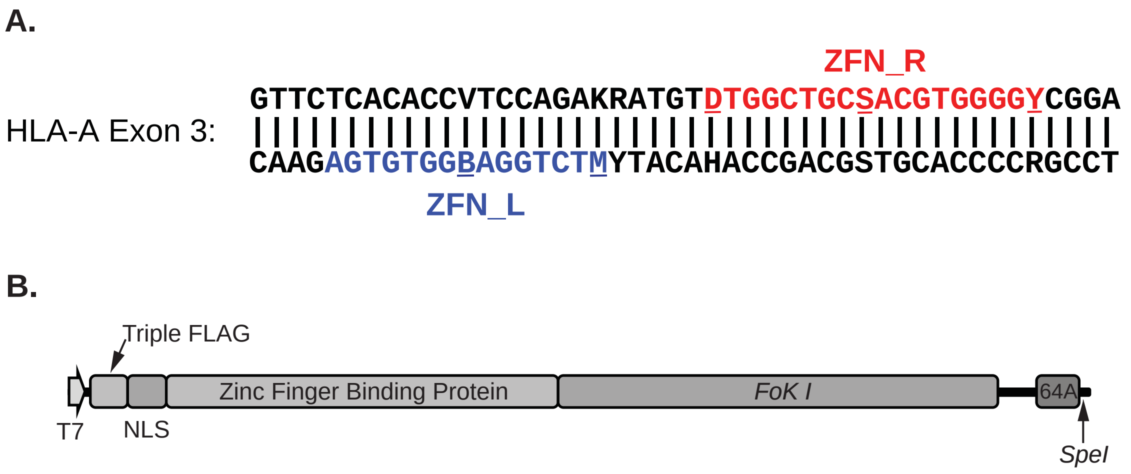

Supplementary Figure 1. HLA-A target ZFN target site and template plasmid for *in vitro* transcription

A. Target sequence of HLA-A target ZFN is shown in red (ZFN_R) and blue (ZFN_L). There are 2 or 3 polymorphic site within target sequence. Nucleotide code used are; V: A/G/C, K: G/T, R: A/G, D: A/G/T, S: G/C, Y: C/T, B: G/C/T, M: A/C, H: A/C/T

B. Schematic presentation of pGEM4z/ZFN/A64. T7: T7 promoter, NLS: Nuclear localization signal, 64A; 64 repeat of adenin

Supplementary Table 1. Antibodies used in this study

Target	mAb clone	Fluorescence	Vendor	Catalogue Number
HLA-A2	BB7.2	PE	BD Pharmingen	558570
HLA-DR	LN3	APC	eBioscience	17-9956-42
CD34	581	APC	BD Pharmingen	555824
CD38	HB7	FITC	BD	340927
CD45	2D1	PerCP.Cy5.5	BD	340953
CD33	WM53	APC	BD Pharmingen	551378
CD14	M5E2	FITC	BD Pharmingen	555397
CD19	HIB19	APC	BD Pharmingen	555415
CD3ε	SK7	FITC	BD	349201
mouse CD45.1	A20	APC	Biolegend	110714

Supplementary Table 2. Population Categories and Registry Sizes

Population	Description	Effective Registry Size (Current Availability)	Total Registry Size (100% Availability)
EURCAU	European Caucasian	3,216,667	6,307,190
AMIND	North American Indian	17,893	63,903
AISC	Amer. Indian South or Central America	2,514	8,977
MENAFC	Mideast/North Coast of Africa	182,211	357,276
KORI	Korean	33,625	115,948
FILII	Filipino	19,822	68,351
NCHI	Chinese	37,469	129,202
CARHIS	Caribbean Hispanic	52,862	182,284
JAPI	Japanese	10,479	36,134
MSWHIS	Mexican or Chicano	136,685	471,326
VIET	Vietnamese	9,483	32,701
ALANAM	Alaska Native or Aleut	650	2,321
SCAHIS	South or Central Amer. Hispanic	62,844	216,704
AINDI	South Asian	73,065	251,949
CARIBI	Caribbean Indian	2,157	7,703
SCSEAI	Other Southeast Asian	10,035	34,605
HAWI	Hawaiian/other Pacific Islander	4,146	14,295
AAFA	African American	140,277	609,899
CARB	Black Caribbean	9.623	41,841
AFB	African	7.982	34,705
SCAMB	Black South or Central American	1,688	7,341

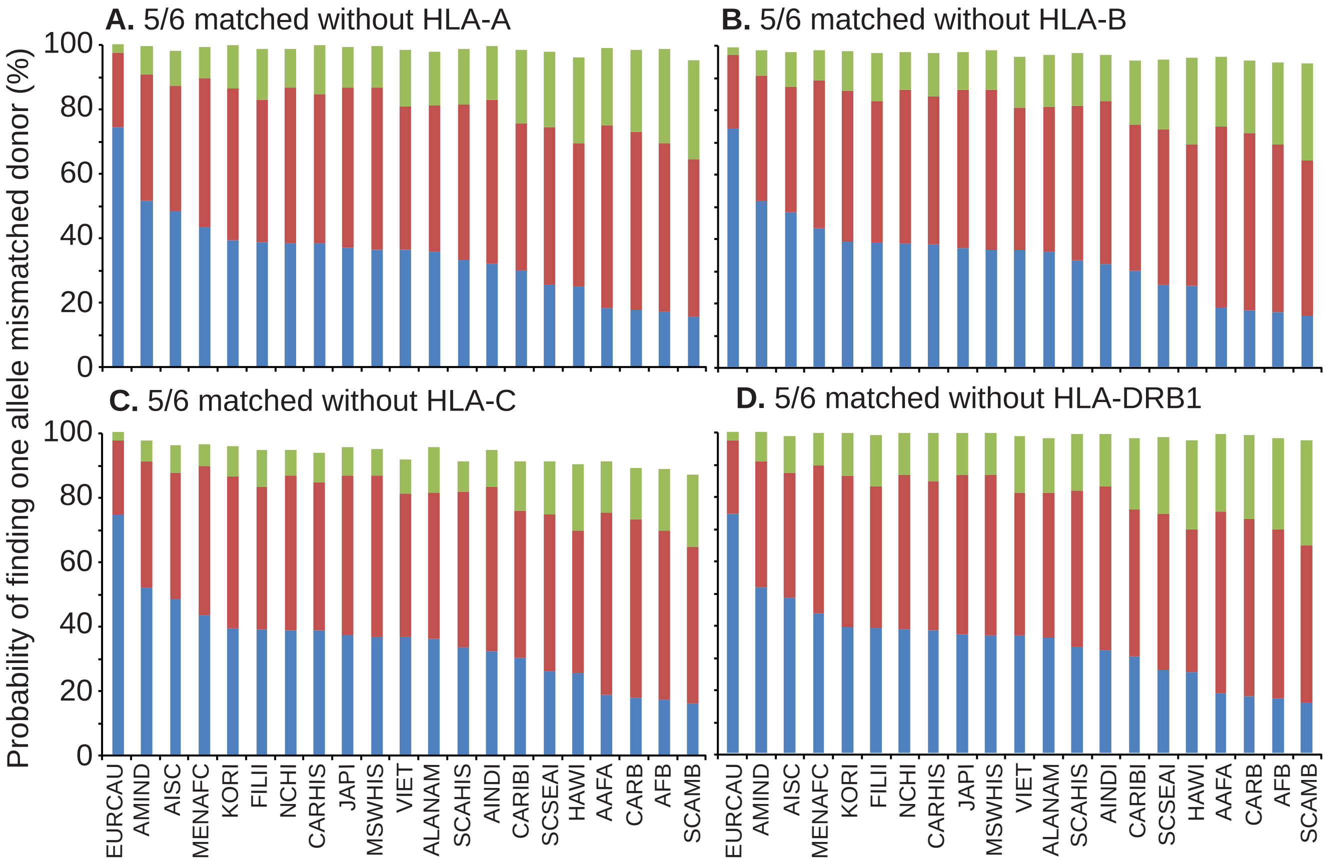

Supplementary Figure 2. Impact of 1 Locus Elimination for Finding One Allele Mismatched Donor in NMDP

Blue bar represents probabilitiy of finding 8/8 matched donor in each race group. Red bar represents probability of finding 7/8 matched donor in each race group. Green bar represents marginal benefit of eliminate designated HLA locus (A. HLA-A, B. HLA-B, C. HLA-C, and D. HLA-DRB1)

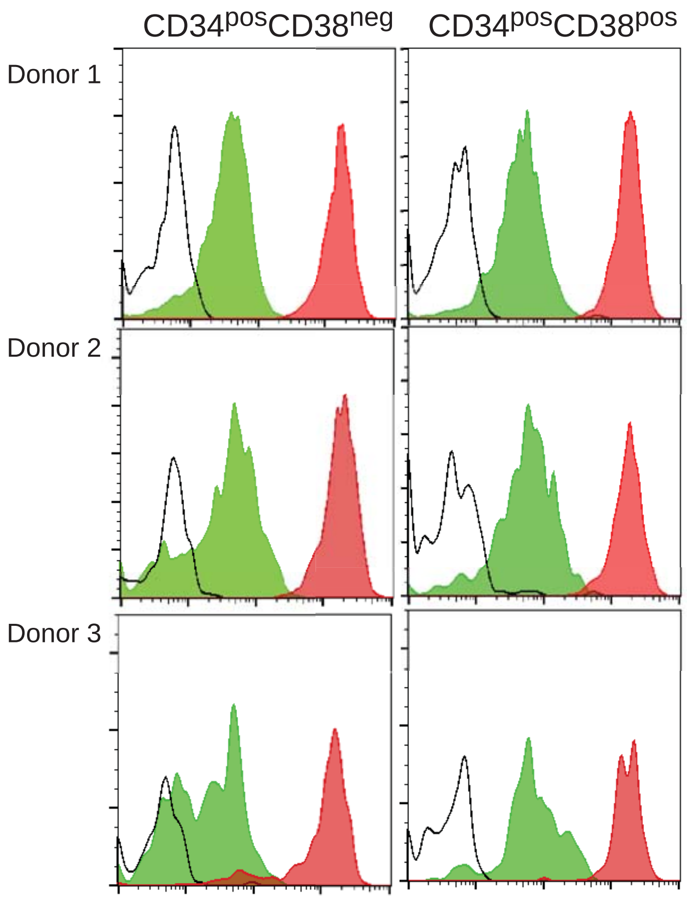

Supplementary Figure 3. HLA class I and class II expression on HSCs

Expression of HLA-class I (red) and HLA-class II (HLA-DR) are shown. Black line represents isotype control

Supplementary Table 3. ELISA based assay to evaluate ZFN binding affinity to specific HLA-A alleles.

Rank in EUR	Frequency in EUR	HLA-A allele	Sequence			Normalized value (AU)	
			ZFN_L binding site	spacer	ZFN_R binding site	ZFN_Left	ZFN_Right
1	0.29604	0201g	TCACACCGTCCAGAG	GATGT	ATGGCTGCGACGTGGGGT	0.7	1.22
2	0.17181	0101g	TCACACCATCCAGAT	AATGT	ATGGCTGCGACGTGGGGC	0.67	1.03
3	0.14347	0301g	TCACACCATCCAGAT	AATGT	ATGGCTGCGACGTGGGGT	0.67	1.22
4	0.08686	2402g	TCACACCCTCCAGAT	GATGT	TTGGCTGCGACGTGGGGT	0.61	1.22
5	0.05642	1101g	TCACACCATCCAGAT	AATGT	ATGGCTGCGACGTGGGGC	0.67	1.03
6	0.03279	2902	TCACACCATCCAGAT	GATGT	ATGGCTGCGACGTGGGGT	0.67	1.22
7	0.03133	3201	TCACACCATCCAGAT	GATGT	ATGGCTGCGACGTGGGGC	0.5	1.03
8	0.02948	2601g	TCACACCATCCAGAG	GATGT	ATGGCTGCGACGTGGGGC	0.26	1.03
9	0.02503	6801g	TCACACCATCCAGAT	GATGT	ATGGCTGCGACGTGGGGT	0.67	1.22
10	0.02351	3101	TCACACCATCCAGAT	GATGT	ATGGCTGCGACGTGGGGT	0.67	1.22

Supplementary Table 4. The descriptions of 47 genes differentially expressed between cells with SR1 and cells without SR1 under both conditions of without ZFN and with ZFN

PROBE_ID	Average Fold Change	Gene ID	Entrez Gene Name	Location	Type(s)
ILMN_1693338	-103.325	CYP1B1	cytochrome P450, family 1, subfamily B, polypeptide 1	Cytoplasm	enzyme
ILMN_1671142	-37.489	GPR68	G protein-coupled receptor 68	Plasma Membrane	G-protein coupled receptor
ILMN_1788538	-32.136	NCALD	neurocalcin delta	Cytoplasm	other
ILMN_1699925	-15.772	TGM5	transglutaminase 5	Cytoplasm	enzyme
ILMN_1759920	-13.372	CTTNBP2	cortactin binding protein 2	Cytoplasm	other
ILMN_1680874	-11.163	TUBB2B	tubulin, beta 2B class IIb	Cytoplasm	other
ILMN_2150856	-8.835	SERPINB2	serpin peptidase inhibitor, clade B (ovalbumin), member 2	Extracellular Space	other
ILMN_2365091	-8.691	FCAR	Fc fragment of IgA, receptor for	Plasma Membrane	other
ILMN_2343097	-8.574	NCALD	neurocalcin delta	Cytoplasm	other
ILMN_2109197	-3.831	EPB41L3	erythrocyte membrane protein band 4.1-like 3	Plasma Membrane	other
ILMN_1692893	-3.526	LOC729486			
ILMN_2103107	-3.206	ADAMDEC1	ADAM-like, decysin 1	Extracellular Space	peptidase
ILMN_1811303	-3.001	NR5A2	nuclear receptor subfamily 5, group A, member 2	Nucleus	ligand-dependent nuclear receptor
ILMN_1700081	-2.964	FST	follistatin	Extracellular Space	other
ILMN_1752728	-2.819	FUCA1	fucosidase, alpha-L- 1, tissue	Cytoplasm	enzyme
ILMN_1669888	-2.784	CSTA	cystatin A (stefin A)	Cytoplasm	other
ILMN_1799387	-2.767	INO80	INO80 complex subunit	Nucleus	enzyme
ILMN_3243366	-2.763	C2orf55	KIAA1211-like	Other	other
ILMN_1723035	-2.753	OLR1	oxidized low density lipoprotein (lectin-like) receptor 1	Plasma Membrane	transmembrane receptor
ILMN_1686116	-2.702	THBS1	thrombospondin 1	Extracellular Space	other
ILMN_1714592	-2.517	CDA	cytidine deaminase	Nucleus	enzyme
ILMN_1776121	-2.415	MGC42367			
ILMN_1814153	-2.288	LOC729486			
ILMN_1763837	-2.281	ANPEP	alanyl (membrane) aminopeptidase	Plasma Membrane	peptidase
ILMN_3307868	-2.147	CHI3L1	chitinase 3-like 1 (cartilage glycoprotein-39)	Extracellular Space	enzyme
ILMN_1694966	-2.109	ASGR2	asialoglycoprotein receptor 2	Plasma Membrane	transmembrane receptor
ILMN_1788886	2.205	TOX	thymocyte selection-associated high mobility group box	Nucleus	other

ILMN_2064725	2.341	METTL7B	methyltransferase like 7B	Other	enzyme
ILMN_1701613	2.36	RARRES3	retinoic acid receptor responder (tazarotene induced) 3	Cytoplasm	enzyme
ILMN_1815346	2.39	TMEM136	transmembrane protein 136	Other	other
ILMN_1724422	2.687	SELL	selectin L	Plasma Membrane	transmembrane receptor
ILMN_1886655	2.728	ILMN_1886655	interleukin 23, alpha subunit p19	Extracellular Space	cytokine
ILMN_1719905	2.986	TLR10	toll-like receptor 10	Plasma Membrane	transmembrane receptor
ILMN_1713751	3.005	ADAM19	ADAM metallopeptidase domain 19	Plasma Membrane	peptidase
ILMN_2120695	3.104	TSPAN7	tetraspanin 7	Plasma Membrane	other
ILMN_2341229	3.142	CD34	CD34 molecule	Plasma Membrane	other
ILMN_1698019	3.148	LGMN	legumain	Cytoplasm	peptidase
ILMN_2332964	3.247	LGMN	legumain	Cytoplasm	peptidase
ILMN_1732799	3.328	CD34	CD34 molecule	Plasma Membrane	other
ILMN_1798360	3.402	CXCR7	atypical chemokine receptor 3	Plasma Membrane	G-protein coupled receptor
ILMN_2105441	4.098	IGJ	immunoglobulin J polypeptide, linker protein for immunoglobulin alpha and mu polypeptides	Extracellular Space	other
ILMN_1685608	4.488	NPTX2	neuronal pentraxin II	Extracellular Space	other
ILMN_1751020	5.818	PACSIN1	protein kinase C and casein kinase substrate in neurons 1	Cytoplasm	kinase
ILMN_1740426	5.969	RASD1	RAS, dexamethasone-induced 1	Cytoplasm	enzyme
ILMN_2325837	12.074	CD3D	CD3d molecule, delta (CD3-TCR complex)	Plasma Membrane	transmembrane receptor
ILMN_1738549	16.134	VPREB1	pre-B lymphocyte 1	Plasma Membrane	other
ILMN_1678170	17.028	MME	membrane metallo-endopeptidase	Plasma Membrane	peptidase

Supplementary Table 5. The gene description of 6 differentially expressed genes between cells without ZFN and cells with ZFN under both SR1 and without SR1 conditions

PROBE_ID	Fold Change	Gene	Entrez Gene Name	Location	Type(s)
ILMN_1668850	-2.904	PCSK9	proprotein convertase subtilisin/kexin type 9	Extracellular Space	peptidase
ILMN_1696512	-2.863	AHSP	alpha hemoglobin stabilizing protein	Cytoplasm	other
ILMN_1777031	-2.511	PKLR	pyruvate kinase, liver and RBC	Cytoplasm	kinase
ILMN_2165993	-7.577	ITLN1	intelectin 1 (galactofuranose binding)	Plasma Membrane	other
ILMN_2380561	-3.06	PKLR	pyruvate kinase, liver and RBC	Cytoplasm	kinase
ILMN_3239254	-2.227	UCA1	urothelial cancer associated 1 (non-protein coding)	Other	other
